# Time to Decide? Simplicity and Congruity in Comparative Judgment

**DOI:** 10.1037/a0037411

**Published:** 2014-07-28

**Authors:** Caren A. Frosch, Rachel McCloy, C. Philip Beaman, Kate Goddard

**Affiliations:** 1 School of Psychology, University of Leicester; 2 School of Psychology and Clinical Language Sciences, University of Reading; 3 School of Informatics, City University London

**Keywords:** simple heuristics, congruity effect, magnitude judgments, response times

## Abstract

What is the relationship between magnitude judgments relying on directly available characteristics versus probabilistic cues? Question frame was manipulated in a comparative judgment task previously assumed to involve inference across a probabilistic mental model (e.g., “Which city is largest”—the “larger” question—vs. “Which city is smallest”—the “smaller” question). Participants identified either the largest or smallest city (Experiments 1a and 2) or the richest or poorest person (Experiment 1b) in a 3-alternative forced-choice (3-AFC) task (Experiment 1) or a 2-AFC task (Experiment 2). Response times revealed an interaction between question frame and the number of options recognized. When participants were asked the smaller question, response times were shorter when none of the options were recognized. The opposite pattern was found when participants were asked the larger question: response time was shorter when all options were recognized. These task–stimuli congruity results in judgment under uncertainty are consistent with, and predicted by, theories of magnitude comparison, which make use of deductive inferences from declarative knowledge.

Two bodies of literature examine relative magnitude judgment: one from a decision-making/heuristic perspective in which judgments under uncertainty result in making choices based upon search through appropriate probabilistic cues retrieved from memory (*probabilistic mental models* or *inference from memory*; Gigerenzer & Goldstein, 1996). For example, when making a judgment about which of two cities is larger and the answer is not known, a person needs to make an inference based on probabilistic cues to largeness, such as whether one is a capital city. The other body of literature, which takes a more psychophysical perspective, typically examines judgments based on directly available characteristics, either physically or in memory (e.g., determining which of two digits is larger or which of two sounds is loudest; Banks, Fujii, & Kayra-Stuart, 1976; Banks & Root, 1979). Both literatures are concerned with judgments of the relative value of items along a quantifiable-criterion scale (for simplicity, we refer to this simply as *magnitude*), and both—potentially—make predictions regarding the speed with which such judgments are made. We first consider the two literatures separately and then discuss how the psychophysically inspired framework used to study such judgments under certainty might be applied to understanding the cognitive processes involved in making judgments under uncertainty. Furthermore, we examine whether response time (RT) effects found using binary judgments (two-alternative forced choice, or 2 AFC) can be found in judgments under uncertainty using a three-alternative forced choice (3AFC) task.

## Inference Under Uncertainty

One of the most influential research programs examining judgments under uncertainty in recent years has been the simple heuristics approach of Gigerenzer and colleagues (Gigerenzer & Gaissmaier, 2011; Gigerenzer & Goldstein, 1996; Gigerenzer, Todd, & the ABC research group, 1999). Simple heuristics are employed to solve problems whenever a probabilistic mental model of the task is constructed to solve a general knowledge question or judgment that cannot be solved by accessing or generating certain knowledge (e.g., by using deductive inference from existing declarative knowledge; Gigerenzer, Hoffrage & Kleinbölting, 1991). For example, the question “If you see the nationality letter *P* on a car, is it from Poland or Portugal?” can be solved either by information that is directly retrievable from memory or by deductive inference from the information within memory (e.g., “*PL* is Poland so it must be Portugal”). In contrast, the answer to the question “Which country has the larger population, Poland or Portugal?” cannot be deductively inferred and is unlikely to be retrieved directly. Instead, a probabilistic judgment must be made.

The task on which simple heuristics have most frequently been tested is the judgment of the “superiority” of an item compared with its competitor along a particular criterion or dimension (Gigerenzer et al., 1999). In practice, this often reduces to a judgment of relative magnitude across the dimension in question. For example, judgments such as which of two countries (Poland or Portugal) or cities (San Diego or San Antonio) has the larger population. This problem can be addressed in multiple different ways, but one of the best known simple heuristics is the recognition heuristic (Goldstein & Gigerenzer, 2002). This heuristic is invoked when the mere recognition of an object is a predictor of the target variable (e.g., recognition of a city name is a predictor of population size). Under these conditions, participants typically infer that a recognized item is more likely to be larger than an unrecognized item. This notion is supported by data showing that direct estimates of magnitudes for unrecognized target items are systematically smaller than those for recognized items (Brown, 2002; P. J. Lee & Brown, 2004; see also Figures 3 and 7 in Schweickart & Brown, 2013). It is also supported by various empirical observations that the recognized item is often chosen over an unrecognized item when paired comparisons are made (Gigerenzer & Brighton, 2009; Goldstein & Gigerenzer, 2002), although the exact reasons for this result are disputed (Hilbig & Richter, 2011; see also McCloy, Beaman, Frosch & Goddard, 2010, and Pachur, Todd, Gigerenzer, Schooler & Goldstein, 2011, for discussions).

The simple heuristics approach in which a single “best” cue (such as recognition) is used to inform judgment is often contrasted with a more traditional view in which all available evidence is integrated to provide an “optimal” weighting of the information available, with the judgment going towards the option which has the majority of the (appropriately weighted) information in its favor. Such opposing views about how information is processed exist within other domains within cognitive psychology too. For example, Triesch, Ballard, Hayhoe, and Sullivan (2003) demonstrated that the visual system is far more frugal in collecting data from the environment than has previously been suggested; people only process visual information “just in time” if the current goal requires it. Similarly, frugal processing has been demonstrated in location memory (Lansdale, Humphries & Flynn, 2013) in that participants memorized locations with reference to one anchor point at the expense of more precise and more costly processing, which would have required two anchor points.

Within the domain of judgment and decision making, direct comparisons of the simple heuristics approach and the optimal weighting approach have given mixed results when based on the choices made by individuals (Gigerenzer & Brighton, 2009; Goldstein & Gigerenzer, 2002; Hilbig & Richter, 2011; M. D. Lee & Cummins, 2004; Newell & Fernandez, 2006; Newell, Weston & Shanks, 2003). An alternative is to use RT data. In comparing different models of judgment and decision making, RT data are invaluable in distinguishing between the cues consulted to inform judgment, particularly if cues are consulted serially and singly. For example, faced with a pair of alternatives that cannot be discriminated by the first few cues in a rank-ordered list of “best” cues (the *take the best* heuristic; Gigerenzer & Goldstein, 1996), individuals using this process naturally take longer to come to a decision than when the very first cue is discriminatory (Bergert & Nosofsky, 2007; Bröder & Gaissmaier, 2007; Pachur & Hertwig, 2006).^1^ For example, if a judgment between two cities is made based on recognition of only one of the cities, judgments should be quicker than if both cities are recognized because additional cues need to be recruited if recognition is not itself a useful cue.

## Inference From Certain Knowledge

As N. R. Brown and colleagues have pointed out (N. R. Brown & Tan, 2011; Schweickart & Brown, in press), binary comparison tasks of the kind employed in the investigation of the heuristics assumed to underlie probabilistic inference under uncertainty were previously investigated—using RT data—by an earlier generation of psychophysically inspired researchers also interested in magnitude comparisons (e.g., Banks, 1977). A key difference between magnitude comparison and simple heuristic accounts of relative magnitude judgment is that heuristics simply return which of the options under consideration is likely to be larger, whereas magnitude comparison accounts include an estimate of overall absolute magnitude (e.g., the comparison process yields information as to whether *both* items are particularly large or small, not just which is larger). In particular, this earlier research program aimed to explain the origin of several well-established RT phenomena, including the symbolic distance effect and the semantic congruity effect (henceforth, the *congruity effect*; Banks et al., 1976), effects that reveal the availability of some (albeit possibly coarse-grained) absolute magnitude information.

The symbolic distance effect refers to the finding that RTs are inversely proportional to the difference between the two compared items on the comparison of interest. Responses to the question of whether the digit 9 is greater than 2 are faster than responses to the question of whether the digit 4 is greater than 2, for example. The congruity effect refers to the finding that large values on a continuum (e.g., number, intensity, luminance etc.) are more rapidly compared when participants are asked, “Which is larger?,” whereas small values are more rapidly compared when participants are asked, “Which is smaller?” In other words, participants are faster to compare two values when their overall magnitude is perceived to be *congruent* with the verbal phrasing of the question.

Research by N. R. Brown and Tan (2011) and by Schweickart and Brown (2013) confirmed a classic symbolic distance effect for decisions where simple heuristics might be considered applicable concerning which of two vehicles was the more expensive (N. R. Brown & Tan, 2011) or the relative gross domestic product (GDP) of two countries, even when one of the countries was unrecognized (Schweickart & Brown, 2013). RTs were slower when the price of vehicles or the GDPs of the countries were closer together, even if highly valid probabilistic cues were available which clearly discriminated between the two options. Thus, problem situations that involved magnitude comparison gave rise to symbolic distance effects regardless of whether they could be solved directly (the classic finding) or by means of a heuristic-based probabilistic inference. These findings were taken as evidence for a magnitude comparison process during which magnitude values are generated or retrieved for each item in the pair prior to a comparison process. This explanation provides an alternative framework to the simple heuristics approach in which the generation of magnitude values per se is not required, and hence a symbolic magnitude effect is not anticipated if appropriate cues to magnitude can be utilized at an early stage (e.g., if the cars are of a different status class—luxury or nonluxury brands—this information could inform a simple heuristic; N. R. Brown & Tan, 2011).

Multiple ways of comparing magnitude are possible within a magnitude comparison account, just as multiple heuristics might—in principle—be applied to a probabilistic mental model. N. R. Brown and colleagues (Brown & Tan, 2011; Schweickart & Brown, 2013) discussed the symbolic distance effect with reference to two-stage magnitude comparison models in which magnitudes are generated and then compared (e.g., the *semantic coding model*, Banks, 1977; and the *scan-plus comparison*, Moyer & Bayer, 1976; Moyer & Dumais, 1978). An alternative model, not discussed by N. R. Brown and colleagues, suggests a single-stage process in which the information retrieved about each item in the pair is compared with an ideal end point (Dehaene, 1989; Holyoak, 1977; Jamieson & Petrusic, 1975). All three models, however, predict both symbolic distance and semantic congruity effects (see Duncan & McFarland, 1980, for a discussion). For simplicity’s sake, we describe only the operation of single-stage models. Nothing that follows is reliant upon a single-stage, rather than a two-stage, magnitude comparison model (such as semantic coding or scan-plus-comparison). In the following series of experiments, we aimed simply to examine whether—in addition to the symbolic distance effects already documented—*congruity* effects can also be obtained under conditions where a probabilistic inference is required and either a magnitude comparison or a simple heuristic process might apply.

## The Congruity Effect in Magnitude Comparison Tasks

Empirically, the congruity effect is well established within the magnitude comparison literature. The “choose smaller” condition is often slower overall than the “choose larger” condition (Dehaene, 1989), but the purest form of the congruity effect—a crossover interaction between question frame and magnitude—has been observed with a diverse range of attributes (Banks & Flora, 1977; Holyoak & Mah, 1982; Jamieson & Petrusic, 1975), including brightness (Audley & Wallis, 1964), loudness (Banks & Root, 1979), or number (Cantlon & Brannon, 2005). These results were obtained under conditions of certainty (the answer is known; e.g., “Is 2 larger than 4?”) where a deductive inference could be made from declarative knowledge rather than under uncertainty (the answer is probably unknown to the participant; e.g., “Is Paris larger than London?”) where a heuristic inference based upon a probabilistic mental model might apply (Gigerenzer et al., 1991). However, in principle, a magnitude comparison process could apply to either situation as shown by N. R. Brown and Tan (2011) and Schweickart and Brown (2013). Our first aim, therefore, was to determine whether congruity effects are observable when any inferences made must be made probabilistically and are not deductively valid.

To account for the congruity effect, single-stage magnitude comparison models assume that the “larger” question sets up a large reference point against which the stimuli are compared (e.g., 9, if the question were about digits, or London, if the question was about U.K. cities). Items close to this external referent can be compared more quickly (are more *discriminable*, in Jamieson and Petrusic’s [1975] terminology). Hence, faster judgments are made if the stimuli are large (close to the referent) than if they are small (far from the referent). Similarly, if the question asks for the smallest item, a “small” external referent (e.g., 0) is established, and hence judgments are faster for small items. Thus, a crossover interaction is observed in the RT data.

As a concrete example, under conditions where a probabilistic mental model might apply, if a pair of items to be compared happen to be the second and third largest cities in Germany (Hamburg and Munich), and the task is to judge which of these two is the largest, then the chosen reference point might be the largest city in Germany (Berlin). From the perspective of Berlin, these cities are easy to discriminate (e.g., when placed in rank order of size, their ratio is 2:3), whereas cities further down the list (e.g., the ninth and 10th largest cities, Düsseldorf and Bremen) are further away from Berlin, and the ratio of the difference is much smaller (9:10 in this case). Hence, the cities are much less discriminable along this dimension, and the judgment accordingly takes longer. Alternately, if the reference point is the 11th largest city (Duisburg) then counting back from this point, the position is reversed: the ratio for Düsseldorf and Bremen becomes 2:3 and the ratio for Hamburg and Munich is 9:10.

This form of ratio comparison is used in recent influential theories of memory and classification (G D A Brown, Neath, & Chater, 2007), which include models of semantic memory (Kelley, Neath & Surprenant, 2013). All that is needed to produce congruity effects, therefore, is the assumption that if asked to judge “Which is larger,” a large reference point (such as Berlin) is chosen, whereas if the question is “Which is smaller,” a smaller reference point (such as Duisburg) is chosen. The choice of reference points dictating later judgments is reminiscent of certain aspects of prospect theory (Kahneman & Tversky, 1979), and like that theory, the framework is mathematical/psychophysical and does not provide the mechanistic detail needed to formulate a process model. We defer consideration of these details until the General Discussion.

In our experiments, we assumed that a set of items that are all recognized would be likely to be viewed, at the time of choice, as a large set (i.e., recognition is taken as a cue for largeness). A set of items of which none are recognized would, in contrast, be viewed as a set of small items, (i.e., lack of recognition is taken as a cue for smallness). Hence, if this assumption is correct, for a congruity effect to be observed, RTs should be faster when participants are asked to indicate the largest of a set of items that are all recognized than when they are asked to indicate the largest of a set of items that are all unrecognized (largeness congruity). When participants are asked to indicate the smallest of a set of items, all of which are unrecognized, then RTs should be faster than when they are asked to indicate the smallest of a set of recognized items (smallness congruity). In other words, there should be an interaction between question frame and number of items recognized. Although the assumption we made about recognition informing magnitude judgments derives from the simple heuristic framework, the congruity effect itself is not predicted by any heuristic we are aware of within this framework but instead is predicted by a magnitude comparison process of the kind outlined earlier, wherein judgment is ultimately based upon forming an overall impression of size rather than merely direct comparison of different cues to size.

In all experiments, we varied the question frame so that participants made judgments requiring them to identify the item with the smallest magnitude as well as the item with the largest magnitude. In Experiments 1a and 1b, participants made judgments in three-alternative forced-choice (3-AFC) questions about city size (Experiment 1a) and wealth (Experiment 1b). These experiments represent a departure from the more usual binary comparison task used exclusively in magnitude comparison studies and almost exclusively in studies of simple heuristics and probabilistic mental models and therefore provide a novel testing ground for RT studies of both frameworks. However, there are good theoretical (Beaman, 2013; McCloy, Beaman & Smith, 2008) and empirical (Frosch, Beaman & McCloy, 2007; Marewski, Gaissmaier, Schooler, Goldstein & Gigerenzer, 2010) reasons to believe that the use of heuristics should generalize to multiple-alternative comparisons, and there is likewise no a priori reason to suppose that magnitude comparisons should not also do so. In Experiment 2, we employed a 2-AFC task with city names to confirm that the novel findings from Experiment 1 are also observed in the type of binary judgments that has been used exclusively in magnitude comparison tasks up until this point. In this experiment, we also addressed the possibility that time to recognize the items might be influencing our results by statistically controlling the recognition times for each name.

## Experiment 1

In Experiments 1a and 1b, we used a 3-AFC approach to simultaneously test two hypotheses: the prediction of a congruity effect that relates to the RTs for situations where either all names are recognized or all names are unrecognized and the prediction that—whenever possible, that is, when some items are not recognized—no information other than “mere” recognition is consulted before the magnitude inference is made (Goldstein & Gigerenzer, 2002). For example, according to the simple heuristics framework, the fastest RTs should appear when only one of the items is recognized and the fact of recognition informs the decision. For 2-AFC, this is true regardless of whether the question asks for the larger or the smaller of the two items, as the two questions are logically equivalent. A more interesting situation emerges when more than two items are presented. For example, when three items are presented and one is recognized, then fast RTs might be anticipated if the task is to identify the largest of the three. The reason for this speed advantage is that when only one item is recognized, the decision maker can quickly identify the largest item by consulting only one cue for one item, namely, recognition. However, when three items are presented and two are recognized but the “smaller” question is posed, then the position reverses—now the anticipated faster responses are associated with a situation in which two items are recognized and one is not. This is because under these circumstances, lack of recognition is the cue relevant to the judgment being made and hence the judgment can be made quickly as one item can be identified with aid of this recognition (or lack of recognition) cue. Thus, if we employed a 3-AFC choice judgment task, then a RT advantage would be predicted across two different situations: for the recognize–1 situation with the “larger” question and in the recognize–2 situation with the “smaller” question. Conversely, a congruity effect would manifest itself as faster RTs if all the items are recognized when the *larger* question is asked (relative to when the smaller question is asked) and faster RTs if none of the items are recognized when the *smaller* question is asked (relative to when the larger question is asked).

To summarize, the hypotheses for this experiment were as follows: First, the recognition heuristic—the idea that recognition is used as the sole cue to inform judgment (Goldstein & Gigerenzer, 2002)—predicts that faster responses should be seen when only one item is recognized and the “larger” question is asked. This is the standard question for most studies of comparative judgment, in which the participant is required to indicate which option is the best along the criterion given (e.g., “Which city is largest”). Faster responses should also be seen, however, when *n* − 1 items are recognized and the “smaller” question is asked (e.g., “Which city is smallest?”; Frosch et al., 2007; McCloy et al., 2010). These hypotheses are based upon the presumption that—all else being equal—a response that requires only a single cue to be consulted (recognition) should be faster than a response that requires multiple cues to be employed. These predictions assume, as per Pachur and Hertwig (2006), that retrieval of the recognition cue precedes retrieval of any other information.

In contrast to the predictions derived from the recognition heuristic that focus on judgments where some items are recognized, the magnitude comparison framework makes specific predictions in circumstances when all items or none of the items are recognized. Accordingly, it predicts an interaction such that RTs are faster when the stimuli and the question are congruent, that is, fast RTs when the “larger” question is asked and all three items are recognized, and the opposite pattern (fast RTs when no items are recognized) when the smaller question is asked. This is a novel prediction based upon the application of notions of congruity to magnitude judgments other than the purely numerical/semantic (Banks & Flora, 1977; Banks et al., 1976; Dehaene, 1989; Holyoak & Mah, 1982) or psychophysical (Audley & Wallis, 1964; Banks & Root, 1979) and where all versus none of the items are recognized. In Experiment 1a and Experiment 1b, we tested these ideas using the same experimental structure but different sets of stimuli and different participants.

### Method

#### Participants

Seventy-five adult volunteers (30 men and 45 women) took part; 40 in Experiment 1a and 35 in Experiment 1b. Their average age was 26 years (range 19–54).

#### Materials and design

The experiment was presented on a PC laptop using dedicated Visual Basic software that recorded the choice that the participant made and the time taken to make the choice. Stimuli were constructed based on a set of real and fictional city names (adapted from Oppenheimer, 2003), which have previously been found to elicit moderately high levels of responding consistent with use of the recognition heuristic (McCloy et al., 2010). For Experiment 1a, participants were presented with 200 triplets of city names. Each triplet was paired with one of two questions: either “Which is the largest?” or “Which is the smallest?” Presentation of the two questions was blocked and counterbalanced. Participants indicated their responses by means of a button press. For Experiment 1b, we selected a range of names from the annually published Sunday Times Rich List (a list of the 1,000 richest individuals in the United Kingdom together with estimates of their actual wealth), and because, at the time of testing, this domain had not been used before, we pretested the names for recognition. Names considered to carry intrinsic clues to wealth independent of recognition (e.g., double-barreled, titled, or otherwise “aristocratic-sounding” names) were excluded from the sample. The names were thus obtained using the same method from the same source as Frosch et al. (2007); hence, the same highly positive correlation between recognition and criterion (wealth) applied (*r* = .73). Participants were presented with the same 120 triplets of names taken from the Rich List—30 triplets were made up of names that were recognized by only few pretest participants, 30 triplets contained one recognizable name (i.e., which was recognized by most pretest participants) and two unrecognizable names (i.e., not recognized by many pretest participants), 30 contained two recognizable names and one unrecognizable name, and 30 contained three recognizable names. Each triplet was paired with one of two questions: either “Who is the richest?” (60 triplets) or “Who is the poorest?” (60 triplets). For the “poorest” question (smaller on the wealth criterion), the recognition-criterion correlation is identical in magnitude to that for the “richest” question (larger on the wealth criterion) but is negative rather than positive.

#### Procedure

Participants were informed that they would be presented with a series of choices about three towns or cities (Experiment 1a) or about three names (Experiment 1b). The choice would be to identify either the largest of the three towns or the smallest of the three towns (Experiment 1a) or to identify the richest of the three names or the poorest of the three names (Experiment 1b). The question was visually presented on the computer screen above the list of options. Participants made their choice by pressing one of three keys on the keyboard representing the left-hand, middle, or right-hand option. Participants were given a maximum of 3 s in which to make their choice. The time available was visually represented to participants by a countdown bar that appeared below the options on the computer screen. Following the final choice, participants were presented with a list of all the towns or cities (Experiment 1a) or names (Experiment 1b) they had seen and were asked to indicate by checking a box next to the name which options they recognized prior to the task. This procedure enabled us to retrospectively identify for each participant which name triples contained recognized and unrecognized names, and hence it was possible to conclude whether judgments were made in accordance with the recognition heuristic.

### Results and Discussion

**Figure 1 fig1:**
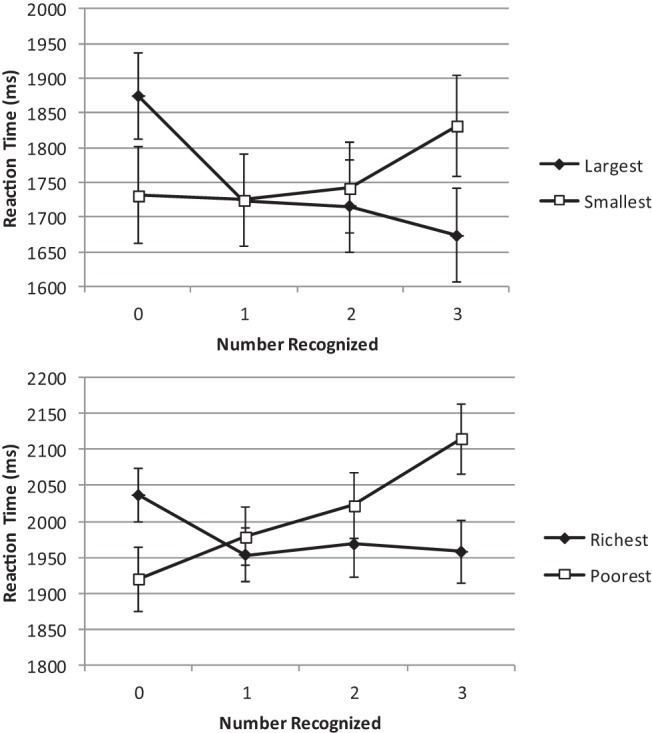
Time to choose as a function of question and number of options recognized. Values are untransformed means values. Bars are standard errors. Experiment 1a (size of cities) is upper panel and Experiment 1b (wealth of people) is lower panel. Median values for key data points in each of these experiments are as follows: Experiment 1a, zero recognition 1,874 ms and 1,731 ms (larger and smaller questions, respectively), full recognition 1,673 ms and 1,831 ms (larger and smaller questions), and Experiment 1b, zero recognition 2,049 ms and 1,925 ms (larger and smaller questions, respectively), full recognition 1,986 ms and 2,193 ms (larger and smaller questions).

We first examined whether recognition was indeed used as a cue to magnitude by ensuring that participants chose recognized names when identifying the largest/richest and unrecognized names when identifying the smallest/poorest. Any congruity effects we find in the RTs can only be interpreted if this prerequisite is met. The number of options recognized was determined individually for each participant based on his or her responses to the final task where they indicated which names they recognized prior to the experiment. Next, we examined the RTs in order to establish whether they were consistent with a congruity effect (fastest RTs for smallest questions when no items were recognized and fastest RTs for largest questions when all items were recognized) or predictions derived from the simple heuristics framework (fastest RTs for smallest questions when only one item was not recognized and fastest RTs for largest questions when only one item was recognized). Figure 1 shows participants’ mean RTs by the number of names they recognized for both Experiments 1a (upper panel) and 1b (lower panel).[Fig-anchor fig1]

#### Recognition as a cue to magnitude

First, we tested whether participants used recognition as a cue to magnitude. For Experiment 1a, participants in the experiment reliably made choices consistent with use of the recognition heuristic. For the larger question, when participants recognized one out of three names, they chose the recognized option significantly more often than at chance, *t*(39) = 9.82, Cohen’s *d* = 1.55, *p* < .001, at a rate of 64%. When they recognized two out of three names, they also chose a recognized name significantly more often than chance, *t*(39) = 10.32, *d* = 1.65, *p* < .001, at a rate of 86%. For the smaller question, when participants recognized one out of three names, they chose the recognized option at approximately chance level (32%), *t*(39) = −0.2, *d* = 0.03, *p* > .05. When they recognized two out of three names, they chose a recognized option significantly less often than chance, *t*(39) = −3.48, *d* = 0.55, *p* < .001, 52% of the time on average.

For Experiment 1b, for the larger question (who is richest), when participants recognized one out of three names, they chose the recognized option significantly more often than at chance, 72% of the time, *t*(34) = 12.60, *d* = 2.13, *p* < .001. When they recognized two out of three names, they also chose a recognized name significantly more often than chance, 87% of the time, *t*(34) = 10.13, *d* = 1.71, *p* < .001. For the smaller question (who is poorest), when participants recognized one out of three names, they chose the recognized option significantly less often than chance, 25% of the time, *t*(34) = −12.19, *d* = 2.06, *p* < .001. When they recognized two out of three names, however, they did not choose a recognized option significantly less often than chance, 39% of the time, *t*(34) = 1.59, *d* = 0.27, *p* = .122. Overall, the choice data for the two experiments indicate that recognition was indeed a cue to largeness (Experiment 1a) and wealth (Experiment 1b) as participants tended to choose recognized options for the largest/richest questions but less so for the smallest/poorest questions.

#### RT data

Next, we considered the time taken to respond to the question posed, in order to test for a congruity effect and examine the predictions derived from the simple heuristics framework. For Experiment 1a, analysis of the time taken to respond using repeated-measures analysis of variance (ANOVA) with question and number of items recognized as within-participant factors found no significant effect of question, *F* < 1, η_p_^2^ = .011, all RT data log-transformed. There was a significant effect of the number recognized, *F*(3, 114) = 5.23, mean square error *(MSE)* = 0.007, η_p_^2^ = .121, *p* = .002, and a significant interaction with question, *F*(3, 114) = 10.62, *MSE* = 0.01, η_p_^2^ = .218, *p* < .001. For the smaller question, participants’ choices were slower as the number of names recognized in each triplet increased. For the “larger” question, the opposite pattern appeared to hold, with participants’ choices becoming faster the more options they recognized (Figure 1, upper panel). With the larger question, there was a significant congruity effect: RTs were significantly faster for the congruent (recognize-all) condition than the incongruent (recognize-none) condition, *t*(39) = 5.28, *d* = 0.68, *p* < .001. With the smaller question, the difference between congruent (recognize none) and incongruent (recognize all) conditions just missed significance, *t*(38) = 1.8, *d* = 0.22, *p* = .07 (both tests two-tailed).

For Experiment 1b, repeated-measures ANOVA likewise found no significant effect of question, *F*(1, 34) = 1.62, *MSE* = 0.007, η_p_^2^ = .046, *p* = .211. There was a significant main effect of the number of names recognized, *F*(3, 102) = 3.70, *MSE* = 0.004, η_p_^2^ = .098, *p* = .014, and a significant interaction between question and response time, *F*(3, 102) = 8.39, *MSE* = 0.007, η_p_^2^ = .198, *p* < .001. For the richer (equivalent to the larger) question, participants’ choices appeared faster when all names were recognized than when there was no recognition, but this result just missed significance, *t*(34) = 1.98, *d* = 0.33, *p* = .056. With the poorer (equivalent to the smaller) question, comparison of no and full recognition was significantly faster for no recognition, *t*(34) = 4.25, *d* = 0.71, *p* < .001, consistent with congruent responding (both tests two-tailed).

The form of the significant interaction shown in Figure 1 is as expected on the basis of a congruity effect predicted by magnitude comparison models. The tendency for participants to choose the recognized item more frequently than chance when asked the larger question (and the unrecognized item more frequently than chance when asked the smaller question) is consistent with the working assumption that participants, implicitly or otherwise, view recognized items as larger than unrecognized items, an assumption that also forms the basis of the recognition heuristic. Thus, the precondition for interpreting the crossover interaction as a consequence of congruity, rather than as a new and previously unconsidered effect, appears to have been met.

Consistent with Hilbig and Pohl (2009), there was no sign that selection consistent with recognition-only inference (the one recognized item in the larger question condition or the one unrecognized item in the smaller question condition) was faster than using knowledge to choose between multiple-recognized items. As Figure 1 shows, numerically at least, participants appear slightly slower on average to choose the single recognized item when asked the larger question. However, like Hilbig and Pohl (2009), this experiment used cities as stimuli, and it is possible that there is something unusual with either this domain generally or these stimuli specifically. In particular, the cities used here were a mix of real and fictional places as was also used by Oppenheimer (2003). Pachur, Bröder and Marewski (2008) criticized the use of these particular fictional materials because they allowed for an informed guess about their country of origin that may have affected the inferences participants made about these. It is also impossible to calculate the validity of recognition as a cue to judgment for fictional items. Thus—although the choice data are consistent with the use of the recognition heuristic—a precondition of employing the recognition heursitic (e.g., Volz et al., 2006) has technically not been met. However, in Experiment 1b, we used different stimuli, a different domain (wealth judgment), and a new pair of questions (richer or poorer), to which these criticisms do not apply. The employment of a set of stimuli, for which verifiable “correct” answers can be provided, also allowed us to estimate the validity of recognition as a judgment cue and the same pattern of data was observed in Experiment 1b as in Experiment 1a. RTs in both experiments follow the same patterns despite the difference in stimuli and choice dimension (city size or wealth).

**Figure 2 fig2:**
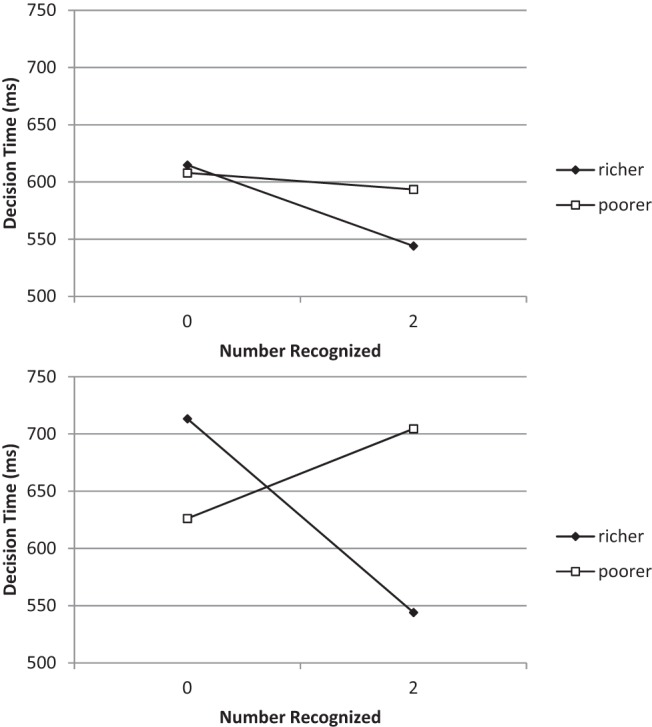
Response time for paired-choice data as a function of question and number of options recognized. Upper panel shows timed-out responses treated as missing data, and lower panel shows the same data with timed-out responses replaced by a maximum response time.

These data pose a challenge for a sequential-step view of the application of simple heuristics. The larger (“Which is largest,” “Who is richest?”) and smaller (“Which is smallest,” “Who is poorest?”) questions reliably produced RT data that are approximate mirror images, one of the other. The crossover interaction, indicating a congruity effect between question and number recognized, predicted by magnitude comparison models (e.g., Jamieson & Petrusic, 1975) was statistically significant. In all cases, with a greater question, the slowest response was to situations where no items were recognized. Fast RTs were observed for situations in which all items were recognized. Conversely, with a lesser question, fast RTs were observed when no items were recognized and RTs were slower when all the items were recognized. Consistent with Hilbig and Pohl (2009), there was no evidence that recognition-only inference was faster when it could potentially be applied (recognized only one item) than when knowledge would necessarily be required on top of recognition (recognize two out of three items; see Figure 2; and for a model of this situation, see Beaman, 2013).[Fig-anchor fig2]

In summary, the results of Experiment 1a and 1b are broadly consistent, indicating that congruity effects occur over at least two domains previously investigated in the context of probabilistic mental models and simple heuristics. However, Experiment 1 used a 3-AFC rather than the traditional 2-AFC task. The question frame manipulation also appeared within a repeated-measures design, and therefore there may be concerns about task-switching costs (Monsell, 2003), even though presentation was blocked, not randomized. Finally, potentially Experiment 1 had problems in the RT measures taken, notably the confounding, within the experimental design, of time to recognize with time to decide. This confound is unlikely to be a major issue as the congruity effect presents as an *interaction* between number recognized and question asked. For the congruity effect to occur, the overall RT should increase as a function of the number of items when one question is asked, *but not the other*. For example, suppose RT increases with the number of items that are unrecognized because of an increase in RT, not decision time. Overall RT would increase as the number of recognized items decreased regardless of question type. This pattern appeared in Experiment 1 only when the smaller question was asked and the opposite pattern occurred when the larger question was asked; hence, a congruity effect was observed. This interaction was not anticipated on the basis of any effect of recognition time on the number of items recognized. Experiment 2 provided a check that the congruity effect is observable within more traditional binary-choice judgments (and is not an unforeseen artifact of expanding the number of items to choose between), addressed the possibility that task-switching may have impacted upon the results of Experiment 1, and provided further evidence that the congruity effect is independent of the (potentially confounding) time to recognize the items.

## Experiment 2

The data from the previous experiments are consistent in showing a congruity effect across the two domains considered in 3-AFC tasks, but there remains a methodological gap between these results and the magnitude comparison literature data showing the same finding. We hope to establish a congruity effect for judgments under uncertainty as for judgments under certainty (e.g., when digit 9 is known to be greater than 5), using domains (city size, individual wealth) previously employed to investigate the application of simple heuristics. However, unlike here, the magnitude comparison literature employed exclusively binary choices. In Experiment 2, we directly examined the possibility of a semantic congruity effect in a more traditional binary (or paired-) choice task. Data from a previous, unpublished, study, carried out when participants were under time pressure, suggested that this would be the case. Figure 2 shows descriptive statistics from this condition both when “timed-out” responses are treated as missing data and when they are assigned a maximum value as in the analysis of that experiment. A funnel-shaped interaction indicative of a congruity effect is evident. Once again, a main effect of number recognized could be dismissed as an artifact of time taken to verify that an item is recognized; the interaction effect which constitutes the expected pattern of congruity results cannot be dismissed in this way.

To continue to examine the possibility of recognition heuristic involvement in these judgments, we employed a multinomial processing tree model, the *r* model (Hilbig, Erdfelder & Pohl, 2010), as a means of simultaneously estimating recognition validity (the *a* parameter in the model) and recognition heuristic usage (the *r* parameter in the model). Where simple choice of a recognized item might be for reasons beyond recognition per se, use of this model allowed us to be more confident in estimating the extent to which recognition itself (rather than recognition plus extra knowledge) is used to inform judgment by finding the best fitting set of parameters for use of recognition and use of knowledge. This also allowed us to examine more closely the use of this heuristic when answering both the larger and the smaller questions (McCloy et al., 2010).

### Method

#### Participants

Our sample was composed of 87 University College London participant panel volunteers who took part in return for a small honorarium. Fourteen participants who recognized five or fewer city names or 16 or more city names were excluded from the analysis, because for those participants there were insufficient data points per condition in one or more of the conditions for the data to be reliable (recall that analysis compares RTs for trials where no items were recognized with RTs for trials where all items were recognized). Three participants who had very extreme RTs^2^ were also excluded. The remaining 70 participants had a mean age of 26 years (range 17–47). There were 32 women and 38 men.

#### Materials and design

We employed a between-participants design in which participants either judged which of a pair of two Canadian cities was smaller or larger. Nineteen Canadian cities were paired to produce 174 pairings^3^ to be judged. The materials were presented in a different random order to each participant. The task was presented using E-Prime software (Schneider, Eschman, & Zuccolotto, 2002) on a desktop PC.

#### Procedure

There was no time limit on making judgments, but participants were asked to make their responses as quickly as possible. They responded by pressing the “a” key to choose the city on the left and the “l” key to choose the city on the right. On completion of the 174 judgments, participants completed a postjudgment recognition task in which each of the city names presented in the experiment was presented one at a time in different random orders, and participants indicated (by pressing the “y” and “n” keys) whether they recognized the cities from before participating in the experiment. RTs from this task were also recorded.

### Results

As for the previous experiment, we begin by ensuring that recognition was indeed used as a cue to magnitude by examining whether the choices were consistent with the recognition heuristic. Next, we examined the RTs in order to establish whether a congruity effect existed for the binary choices made in this experiment. Finally, we also applied a multinomial processing tree model intended to measure the extent to which the recognition heuristic was used *without reference to other knowledge* in order to make the appropriate choices.

#### Recognition as a cue to magnitude

In both conditions, participants made a choice consistent with the recognition heuristic significantly more often than chance. For the larger question, they chose the recognized option 85% of the time; one sample *t* test, *t*(35) = 16.32, *d* = 2.72, *p* < .001, and for the smaller question, they chose the unrecognized option 73% of the time; *t*(33) = 5.9, *d* = 1.01, *p* < .001. Hence, the prerequisite for interpreting a congruity effect is met. Consistent with McCloy et al. (2010) participants in the larger condition made choices consistent with the recognition heuristic significantly more often than participants in the smaller condition; *t*(51.91) = 2.78, *d* = 0.66, *p* = .008.

#### RT data

**Figure 3 fig3:**
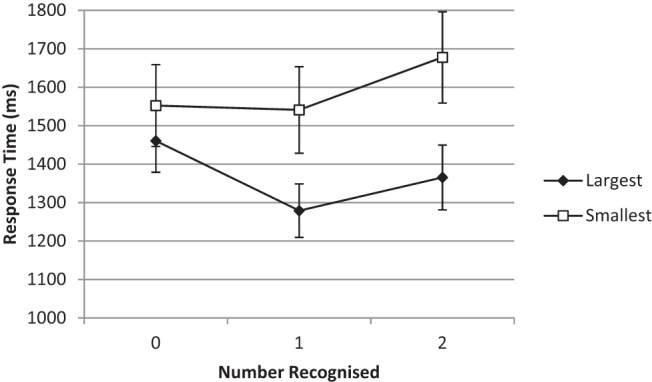
Experiment 2: Time to choose as a function of question and number of options recognized. Bars are standard errors. Median values for key data points are as follows: zero recognition 1,409 ms and 1,510 ms (larger and smaller questions, respectively), full recognition 1,269 ms and 1,447 ms (larger and smaller questions).

Figure 3 shows the mean RTs for number of names recognized by condition. A repeated-measures ANOVA on the RTs for the conditions of either full or zero recognition only with question frame as a between-participants factor revealed no main effect of number recognized; *F*(1, 47) < 1, *MSE* = 0.113, η_p_^2^ *=* .003, *p* = .21, but a main effect of question type; *F*(1, 47) = 18.97, *MSE* = 0.05, η_p_^2^ *=* .288, *p* < .001. There was also a significant interaction between number recognized and question, *F*(1, 47) = 12.11, η_p_^2^ *=* .205, *p* < .001.^4^ The purest form of congruity effect, a crossover interaction, is not evident in Figure 3, although Dehaene (1989, p. 558) noted that the congruity effect is, in practice, often superimposed on top of other effects that may preclude a full crossover effect and instead often appears as a funnel-shaped interaction between instructions and the size of the stimuli (large or small) where an advantage for the larger question is more likely to be in evidence than for the smaller question.[Fig-anchor fig3]

Once again, we compared the RTs for the items where participants recognized none of the names with RTs for the items where they recognized all of the names. In the smaller condition, the participants were quicker to respond to the items when they did not recognize any of the names (mean 1,552 ms) than when they recognized both names (mean 1,677 ms), *t*(33) = 2.01, *d* = 0.31, *p* = .05. In the larger condition, the participants responded more quickly to the items where they recognized both names (mean 1,365 ms) than to the items where they recognized none (mean 1,460 ms), *t*(35) = 2.1, *d* = 0.20, *p* = .03. Note that, for the smaller condition, this numerical difference between the untransformed means in favor of the larger question is reversed by log-transformation. There was no difference between the mean RTs for the two question types when neither was recognized, *t*(68) < 1, but there was a significant difference between the two question types when both were recognized, *t*(68) = 2.17, *d* = 0.5, *p* = .03.

#### Controlling for recognition time

To investigate the contribution of the time taken to recognize the items to the overall response times, we followed a procedure similar to that used by Hilbig and Pohl (2009). Accordingly, we calculated a separate multiple regression for each participant where we predicted RTs for each trial of the judgment task from the RTs for each item in the pair taken from the postjudgment recognition task. We then calculated revised means from the unstandardized residuals generated by the regression analyses. These revised means for trials where either none or all items were recognized were then entered into a repeated-measures ANOVA with question frame as between-subjects factor. This analysis resulted in the loss of the significant main effect of question type, *F*(1, 66) < 1, *p* = .522, but crucially the interaction remained significant, *F*(1, 66) = 4.89, *MSE* = 345,863, η_p_^2^ = .069, *p* = .031.^5^ Planned comparisons between the two question frames revealed a significant difference when none of the items were recognized, *t*(66) = −2.64, *d* = 0.65, *p* = .01, but no difference when both of the items were recognized, *t*(66) = 0.78, *d* = 0.19, *p* = .44. The differences between recognizing none of the items and recognizing all of them within each of the question frames were no longer significant, *t*(33) = −1.86, *d* = −0.35, *p* = .071 for the smaller condition, and *t*(33) = 1.2, *d* = 0.21, *p* = .24 for the larger condition. Hence, when controlling for recognition time, we still observed the interaction between question frame and number of items recognized, and this interaction appeared to be driven by differences found when none of the names were recognized.

#### Recognition and knowledge

**Figure 4 fig4:**
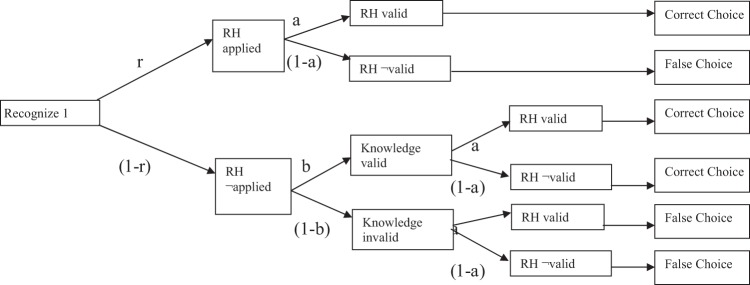
Decision tree for the *r* model when the recognition heuristic is applicable. RH = recognition heuristic; dash (–) = not; *r* = probability that recognition is used; *a* = probability of a correct choice if the recognition heuristic is used; *b* = probability of a correct choice if both options are known.

For this set of data, we also applied a multinomial processing tree model intended to measure the extent to which the recognition heuristic was used *without reference to other knowledge* in order to make the appropriate choices. Alongside this, we also used the discrimination index measure of Hilbig and Pohl (2008), another measure intended to give some indication of the extent to which recognition is used as the sole cue to decision making. The discrimination index is calculated as the proportion of times the recognition heuristic is used when it is valid, less the proportion of times it is used when it is invalid. Multinomial processing tree models assume sequential, independent operations that can be expressed in terms of a decision tree, with alternative processes at each branch point associated with a parameter indicating the probability of traversing that particular branch. The tree terminates in observable outcomes, and the models are compared with the data by estimating the best fitting parameters and comparing the frequency counts of each outcome, obtained from experimental data, with the expected outcomes given the parameters estimated. For a 2-AFC situation to which the recognition heuristic could potentially be applied, there are four free parameters that must be estimated: *g* is the probability of a correct guess if neither of the options are recognized, *b* is the probability of a correct choice if both options are known and should be closely related to the knowledge validity of the domain, *a* is the probability of a correct choice if the recognition heuristic is used and reflects the recognition validity of the domain, and *r* is the probability that recognition is used as the sole cue for inference. Thus, there are three decision trees arising from this situation: when both items are recognized, the probability of a correct outcome is *b* and of an incorrect outcome is 1 − *b*. Similarly, for when none of the items are recognized, probability correct is *g* and probability incorrect is 1 − *g*. The more interesting possibilities (shown in Figure 4) occur when only one item is recognized and recognition can be used as the sole cue to judgment (or not) and, if not, the unrecognized option could conceivably be chosen and, of course, the outcome can be a correct choice (or not).[Fig-anchor fig4]

The results of fitting the *r* model using Moshagen’s (2010) multitree software indicate that the best fitting parameters are *a* = .85, *b* = .68, *g* = .53, and *r* = .54. This model gives a good fit to the data, *G*^2^ = .03, *df* = 1, *p* = .86. The best fitting parameters indicate that for this domain, recognition (*a*) provides a more accurate cue than knowledge (*b*), but, despite this, recognition is used as the sole cue on little more than 50% of occasions. This general impression that recognition is not the sole cue used is confirmed by the discrimination index (DI) where DI = .48. These data refer to the larger question.

Other research (McCloy et al., 2010) shows that the recognition heuristic is employed less frequently when the smaller question is asked. This previous research relied upon relatively insensitive measures of adherence to the heuristic wherein a response was scored as adhering to the heuristic when the greater question was asked and a recognized item was chosen. In fact (as Figure 4 shows) recognized items might be chosen for reasons other than recognition per se (e.g., a town might be recognized for a particular reason, which leads to confidence that it is a large town). In the current data set, the probability that a recognized item was chosen was .85, whereas the best fitting *r* parameter was .54 (in line with the range observed in past research; Beaman 2013; Hilbig, Erdfelder, et al., 2010; Hilbig & Richter, 2011). This implies that approximately 30% of the time the recognized item was chosen for reasons beyond “mere” recognition. Thus, any reduction in recognition heuristic-consistent choices in the smaller question condition here and in McCloy et al.’s (2010) study could have been for reasons unconnected with the recognition heuristic. Hilbig, Scholl, and Pohl (2010) applied the necessary *r* model analysis to similar data—where a smaller question (addressing city population size) produced a nonsignificant trend towards less reliance on the recognition heuristic than a larger question. However, in their report, they identified concerns about the cross-experimental comparisons that they conducted and that limited the conclusions they were able to draw.

Application of the same procedures to the smaller question yielded DI = .60 and the *r* model revealed the following best fitting parameters, *a* = .82, *b* = .67, *g* = .53, and *r* = .14. All of these parameters, with the exception of *r* which we expected to be lower, were within .03 of the values estimated for the larger question. Despite this result, the model fit on this occasion was considerably poorer, *G*^2^ = 3.82, *df* = 1, *p* = .05. This was also true for Hilbig, Erdfelder, et al. (2010), who reported *p* values for differences between observed and expected data dropping from .80–.99 in Experiment 1 (the larger question, *G*^2^ = 0.1) to .29–.32 in Experiment 2 (the smaller question; *G*^2^ = 1.1–14) although in their case the model fits remained within the conventional bounds of statistical acceptability.

One possible reason for the discrepancy in *r* model results is that knowledge validity when two items are recognized (*b*_2_) need not be identical to knowledge validity when one item is recognized (*b*_1_). When both are recognized, it is necessary to distinguish between two known items, whereas when one is recognized the task is to determine whether the known item is of larger or smaller magnitude than an unknown option (see Beaman, Smith, Frosch & McCloy, 2010, p. 265 for a more analytical argument). In practice, it appears that b_1_ is approximately equivalent to b_2_ with the greater question, so both can be subsumed into a single performance parameter, but this is an accuracy measure, not tied to any particular mechanism. The effect of knowledge validity on model fits is likely to be particularly pronounced when the way in which knowledge is applied varies, which, as argued by McCloy et al. (2010), may be the case with the smaller question. Accordingly, we retested the model allowing two knowledge parameters (*b*_*1*_ and *b*_*2*_) but constraining *g* to equal .53 (as the best fitting parameter value estimated from the larger question) since this ensures equivalent *df* and the value of the guessing parameter is of no theoretical consequence provided it is in the region of chance.^6^ Under these constraints, the best fitting free parameters are *a* = .82 (as previously), *b*_*1*_ = .66, *b*_*2*_ = .69, and *r* = .12. This yielded an improved fit to the data, *G*^*2*^ = .01, *df* = 1, *p* = .91. So, by both *r* and DI measures and regardless which version of the *r* model is employed, knowledge is being used much more with the smaller question than the larger question. It seems likely that knowledge is also being used in a different way for the smaller question given the difference in fits between the models.

## General Discussion

The foregoing series of experiments have revealed two main findings. The first finding is that judgments, in Experiment 1, were no faster under conditions where only one item was recognized (or in the case of the lesser question, only one item was not recognized) than when all items were recognized (or in the case of the lesser question, none of the items were recognized). This is inconsistent with the operation of a recognition heuristic alone, where recognition precedes access to other information (Mandler, 1980) and renders the search for further, discriminating information unnecessary (Goldstein & Gigerenzer, 2002; Pachur & Hertwig, 2006). The second main finding is that RT data indicate that the time taken to respond to lesser and greater questions produce approximate mirror images one of the other when plotted against the number of options recognized. This finding is not predicted by the stepwise application of simple heuristics only. It is also not an obvious prediction of many compensatory decision-making procedures (such as multiple regression or structural equation modeling), which take into account and weight multiple sources of information about the options only and do not consider the effects of possible reference points set up by the framing of the question. The finding is predicted, however, by magnitude comparison models, which make use of a reference point established independent of the items under consideration.

The establishment of a congruity effect on choices—particularly paired-choices (Experiment 2)—made under uncertainty adds to the data reported by Brown and Tan (2011) and Schweickart and Brown (2013) showing a symbolic distance effect and provides further support for the idea that a magnitude comparison process (single- or dual-stage) may occur under circumstances previously considered to test heuristic decision making. In contrast to N. R. Brown and colleagues, we have chosen to present single- rather than dual-stage magnitude comparison accounts as the single-stage magnitude comparison models that involve comparisons relative to an end point are conceptually similar to recent and intriguing accounts elsewhere in the literature of discrimination between two or more options in perception, memory, and decision making as a ratio score relative to some other external referent (where the reference point may be drawn from memory but is external to the stimuli under consideration; G. D. A. Brown et al., 2007; Stewart, Brown & Chater, 2005). Both single- and dual-stage magnitude comparison models can account for the current data set, however.

These data suggest that similar processes may be involved in judgments under uncertainty (previously considered the domain of probabilistic mental models and simple heuristics) to judgments where the answer can be directly retrieved or calculated (considered to involve deductive inferences from declarative knowledge). Before any strong conclusions can be drawn from this suggestion, however, it is necessary to consider alternative explanations.

**Figure 5 fig5:**
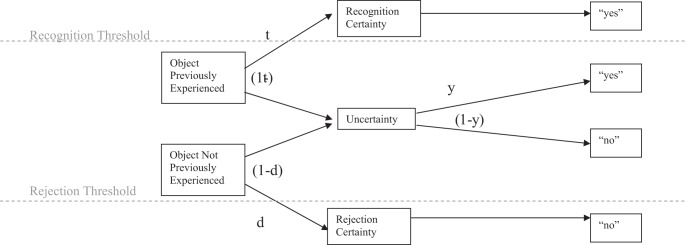
Diagrammatic representation of the memory state heuristic of Erdfelder, Küpper-Tezel and Mattern (2011) as it would appear as multinomial processing tree model. This heuristic predicts faster responses as the number of items in either of the certainty states increases but does not predict an interaction between certainty state and question frame. *t* = probability of old objects exceeding the recognition threshold; *d* = probability of new objects falling below the rejection threshold; *y* = conditional probability of guessing yes in the uncertainty state.

One possible alternative account arises from Erdfelder, Küpper-Tetzel and Mattern’s (2011) mental state heuristic. Erdfelder et al. noted that the single threshold for recognition acceptance assumed in applications of the recognition heuristic might be unrealistic. Instead, they argued, a dual threshold exists both for accepting and rejecting an item as “recognized.” Such an item can be recognized (or rejected) with certainty, or it can recognized (or rejected) rather more hesitantly (see Figure 5).[Fig-anchor fig5]

It is plausible that definite, or certain, decisions (either to recognize or reject) should be faster than more hesitant decisions. Such an account predicts a change in RTs as the number of items subject to definite decisions (in either direction) increases. Thus, a significant effect of number of items recognized would be expected—as the number of items definitely recognized increases, then RTs should decrease, and as the number of items definitely rejected increases, then RTs should likewise decrease. Thus, complete recognition (of all items) should be a relatively fast response condition, and complete failure to recognize any item should also be a fast response condition. This observation complicates the situation with respect to whether the observed response times are incompatible with the recognition heuristic (where one item only is recognized, RTs might be slowed even if the heuristic is employed because the recognition may not be definite; see also Pachur, 2011). The observation does not, however, bear upon the congruity effect because, by this account, all responses in the “recognize-all” condition should be faster—the semantically incongruent ones (“Which item is the smaller?”) as well as the congruent (“Which item is the larger?”). Similarly, the account predicts that responses in the “failure-to-recognize” condition should also all be faster regardless of the question asked (so larger question responses should be speeded as well as smaller question responses), whereas the current data show an interaction between recognition condition and question that is not anticipated by Erdfelder et al.’s (2011) hypothesis.

A number of other conclusions can also be drawn from the current data. The *r* model is particularly informative in this regard. It confirms McCloy et al.’s (2010) findings that recognition per se is used less frequently as a cue when the smaller question is asked. The model also indicates that—at least with the greater question—the heuristic is used for a substantial number of choices (best fitting estimate of *r* = .54). Thus, Schweickart and Brown’s (in press) claim that the adoption of a magnitude comparison approach obviates choosing between competing heuristics may be premature. The magnitude comparison approach also has difficulty in accounting for the framing effect observed by McCloy et al. (2010) (and confirmed here) that the smaller question elicits fewer recognition-based responses than the larger question. In a magnitude comparison framework, both questions should be answered in the same manner by estimating the magnitudes (according to the assumptions of single- and dual-stage models) and making a simple comparison. Thus, although the magnitude comparison framework predicts the RT data reported here, neither it nor the simple heuristics approach as yet fully account for the framing effect observed on choices when logically equivalent questions are asked. A further problem for the magnitude comparison framework is the lack of a well-defined process model. Here, we have favored the single-stage model, but the data are equally compatible with two-stage models as presented by N. R. Brown and colleagues (Brown & Tan, 2011; Schweickart & Brown, 2013). One means of attempting to provide a coherent overall framework for judgments of this type would be to develop a process model in which similarity between the options presented and a reference point was calculated either in a holistic manner (requiring combination of available information in a compensatory manner) or along particular dimensions independently, in a manner more akin to either single-reason heuristic decision making or evidence accumulator models. The development of such process accounts of single-(and dual-) stage magnitude comparisons is necessary to provide a unified account of heuristic-like decision making within a magnitude comparison framework.

Overall, the data suggest that models of comparative judgment developed for comparing physical (e.g., loudness or brightness) or symbolic (e.g., between digits) distances can be successfully applied to judgments that also require a search for appropriate cues from which to infer magnitude. This highlights the usefulness of searching “older” literatures for theories and data that may be applicable to “newer” problems—a point that has been made elsewhere (e.g., Gigerenzer, 1991)—and adds to the data already provided by N. R. Brown and Tan (2011) and Schweickart and Brown (2013), consolidating the general position outlined in those articles. The approach taken here also reinforces the implicit point of G. D. A. Brown and colleagues that psychophysical approaches (here, from magnitude comparison, and in their studies from relative judgment and satisfaction ratings) may be generalized outside the psychophysicist’s laboratory (Boyce, Brown, & Moore, 2010; G. D. A Brown, Gardner, Oswald, & Qian, 2008). More specifically, a congruity effect can be added to the symbolic distance effect as phenomena to be accounted for in *n*-AFC or paired-choice decision tasks. In all cases, it appears, the direction provided by the question framing influences the time required to distinguish between options.

## Footnotes

1 Some accumulator models, where one item has strong evidence, may make similar predictions (see Teodorescu & Usher, 2013, for a review).

2 Two participants had very low RTs throughout (one had a mean of 437 ms [*SD* = 243], and the other had a mean of 386 ms [*SD* = 411]). A third person had a very high mean of 3,810 ms (and a very large SD of 7,866).

3 We intended to include 20 Canadian cities but due to an administrative error the 20th city (Montreal) was only paired with two of the other cities (Toronto and Vancouver).

4 Where the RTs for the incomplete recognition condition are included, the corresponding statistics are *F*(2, 136) = 4.85, *p* = .01, for the main effect of number recognized; *F*(1, 68) = 3.0, *p* = .09, for the main effect of question; and, *F*(2, 136) = 4.41, *p* = .01, for the interaction.

5 Where the RTs for the incomplete recognition condition are included, the corresponding statistics are *F*(2, 132) = 10.31, *p* > .001, for the main effect of number recognized; *F*(1, 66) > 1, *p* = .786, for the main effect of question; and, *F*(2, 132) = 2.5, *p* = .086, for the interaction.

6 Note that setting the value of *g* at .53 might reflect a sampling error since—if we have chosen stimuli such that there are no valid cues associated only with the names of an otherwise unknown pair of items, and performance is therefore at chance in this condition—we would expect *g* to equal .50. Accepting this possibility means that any model that fits the data with *g* wrongly set at .53 must be accurate in other respects, however, as *g* constrains the degrees of freedom available but does not impact upon the estimation of any other values (it only appears within a single decision-tree).
